# Measurement of Salivary Resistin Level in Patients with Type 2 Diabetes

**DOI:** 10.1155/2012/359724

**Published:** 2012-08-30

**Authors:** Jinhua Yin, Hongfei Gao, Jing Yang, Lu Xu, Ming Li

**Affiliations:** ^1^Endocrine Key Laboratory of the Ministry of Health, Department of Endocrinology, Peking Union Medical College Hospital, Chinese Academy of Medical Sciences, No. 1 Shuaifuyuan, Wangfujing, Beijing 100730, China; ^2^First Affiliated Hospital, Shanxi Medical University, Taiyuan 030001, China

## Abstract

Serum resistin was initially hypothesized as a link between obesity and insulin resistance in mice. The latest evidence suggests that serum resistin is proinflammatory cytokines. Inflammation plays a key role in the pathogenesis of type 2 diabetes mellitus (T2DM). Many reports have previously identified changed serum resistin levels in patients with T2DM, but little is known of the levels of resistin in saliva. In our study, saliva and serum samples were collected from 38 patients with newly diagnosed T2DM at each time point of OGTT and 35 nondiabetic controls at fasting state. Resistin concentrations were measured using ELISA. We have demonstrated the presence of resistin in saliva of T2DM and nondiabetic subjects. Saliva resistin levels of T2DM are significantly higher than those of nondiabetic controls. Resistin levels in saliva are not affected by eating activity and correlated with serum resistin levels at any time points of OGTT. A positive correlation of serum and salivary resistin with BMI and HOMA-IR existed in T2DM. Measurement of resistin in saliva is a simple, noninvasive and may be an acceptable alternative to blood sampling for evaluatinginflammation/obesity/insulin resistance state.

## 1. Introduction

Resistin is peptide hormone produced by adipocytes and macrophages. It was originally proposed as the link between obesity and diabetes in mice [1]. In addition, resistin was found to be an in vitro antagonist of insulin on human preadipocytes [2]. Human hepatic cells overexpressing resistin had impaired glucose uptake and glycogen synthesis [3]. The latest evidence suggests that resistin is proinflammatory cytokines [4]. It was positively correlated with proinflammatory factors in adults with pathophysiological conditions such as atherosclerosis, renal disease, inflammation of respiratory tracts, and type 2 diabetes mellitus [5–8].

Human saliva mirrors the body's health and can be collected noninvasively, does not require specialized skills and is suitable for large population-based screening programs [9]. Cortisol levels in saliva at 0:00 are detected to diagnose Cushing's syndrome. Salivary endothelin concentrations are assessed chronic heart failure [10, 11]. Currently, it is reported that adipokines, such as adiponectin, leptin, resistin, and visfatin, can be detected in saliva of healthy subjects [12–14]. To the best of our knowledge, no data on saliva resistin levels in T2DM patients are available at present. The aim of this study was to measure and compare the saliva resistin and serum resistin levels in newly diagnostic T2DM patients and to evaluate whether the saliva and serum levels are correlated. Salivary resistin may be used as a tool to evaluate insulin resistance and inflammatory state for T2DM patients.

## 2. Materials and Methods

### 2.1. Subjects

From June to November 2011, 38 patients (18 males/20 females) with newly diagnosed T2DM and 35 cases (18 males/17 females) of non-diabetes mellitus, who underwent a medical examination at the First Hospital of Shanxi Medical University, Health Screening Center, enrolled in this study. Our local Research Ethics Committee approved the studies, and written informed consent was received from all participants. T2DM group and non-diabetes mellitus control group were matched in male/female ratio and mean age. 

It is noted that, before our medical examination, none of these participants suffered from endocrine malignant or chronic inflammatory diseases or severe systemic illnesses or any recent weight change. None were taking medications including oral contraceptives. In the medical examination process, T2DM was defined according to American Diabetes Association set in 2010. That is, A diagnostic cut point of ≥126 mg/dL (7.0 mmol/L) for FPG and ≥200 mg/dL (11.1 mmol/L) for 2 h PG [15]. 

### 2.2. Measurements

#### 2.2.1. Anthropometric Measurements

We designated a person to anthropometric measurements. Body weight was measured to an accuracy of ±0.2 kg with a standard scale, dressed only with very light clothing, and height was measured to an accuracy of ±0.5 cm using a height bar fixed on the wall, with subjects standing with back, buttocks, and heels pressed together against the wall. Waist circumference was measured to the nearest 0.1 cm at the narrowest part of the torso as seen from the anterior aspect. Hip circumference was measured to the nearest 0.1 cm at the point of maximum extension of the buttocks. Blood pressure was measured twice using a standard mercury manometer, with the participants seated. BMI was computed as weight (kg) divided by squared height (m^2^). Waist-hip ratio (WHR) was computed as waist circumference (cm) divided by hip circumference (cm).

#### 2.2.2. Samples Collection

Whole blood and saliva were obtained before and one, two, and three hours after intake of 75 g glucose in T2DM patients. Only fasting serum and saliva samples were obtained in non-diabetes mellitus. Blood sugar was timely detected at each time point. We also detected HbA1c and liver and kidney function at the fasting state. For serum true insulin and resistin assay, the extracted blood samples were then centrifuged at 1100 g for 10 min. Serum was separated immediately and stored at −80°C until assay was performed. All saliva samples collected by using S-Monovette (Sarstedt, Germany) in 10min were used for this study after being dentally assessed and rinsing their mouths with water thoroughly. Immediately after collection, saliva samples were centrifuged for 10 min at 1100 g, and the supernatant serum was stored at −80°C until analysis of resistin level. Blood and saliva sampling were taken simultaneously but saliva sampling took 5 min much time to collect. Salivary flow rate [16] was computed as volume (mL) divided by time (min). Blood and saliva sampling were measured in the same experimental set.

#### 2.2.3. Sample Detection Methods

Insulin was measured by monoclonal antibody-based sandwich enzyme-linked immunosorbent assay (ELISA), which was developed in the Key Laboratory of Endocrinology, Peking Union Medical College Hospital. The assay had a detection limit of 0.8 mIU/L, covered a working range of 1.5~160.0 mIU/L, and did not cross-react with high dosage of human proinsulin (2000 pmol/L) and C peptide (5000 pmol/L). The mean recovery was 101.7% and the intraassay coefficients of variation and interassay coefficients of variation were below 4.1% and 7.0%, respectively [17].

Glucose concentrations were determined by using the glucose-oxidase-derived technique. Insulin resistance index was calculated by homeostasis model assessment of insulin resistance (HOMA-IR) as (fasting insulin mIU/L) × (fasting glucose mmol/L)/22.5 [18].

Resistin was determined by enzyme immunoassay (EK-028-36, Phoenix Pharmaceuticals Inc., USA). The standards used contained 0, 0.016, 0.031, 0.062, 0.125, 0.25, 0.5, or 1 ng/mL. Intra- and interassay coefficients of variation were below 3.0% and 10%, respectively. Serum samples were diluted with assay buffer at 1 : 20 and salivary samples at 1 : 5 [19].

### 2.3. Statistical Analysis

Descriptive statistics and analysis were performed in SPSS 13.0 for Windows (SPSS Inc. IL). Before statistical analysis, normal distribution was tested. Results are displayed as mean values ± standard deviation. Data of nonnormal distribution were described using the median (M) and quartile (Q1~Q4). The relationships between variables were analyzed by unpaired *t*-test, *repeated measures *ANOVA, and simple correlation (Pearson's test). Correlations with a critical value of *P* < 0.05 were considered significantly.

## 3. Results

### 3.1. Comparative Analysis 

**Figure 1:**
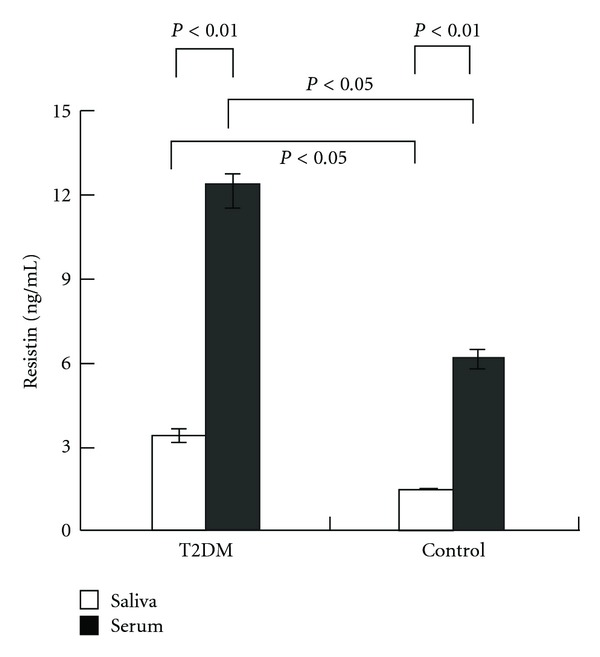
Fasting serum and salivary resistin levels in patients with T2DM and the control.

The difference of age, sex distribution, blood pressure, and fasting salivary flow rate between newly diagnosed T2DM group and control group was not statistically significant (*P* > 0.05). BMI, WHR, HbA1c, fasting glucose, fasting insulin levels, HOMA-IR, serum and salivary resistin levels of T2DM patients were higher than those of nondiabetes mellitus (*P* < 0.05) (Table 1, Figure 1).

Either in diabetic patients or nondiabetes mellitus, resistin can be detected in saliva. Both in control group and T2DM group, resistin levels in serum were significantly higher than those in saliva (*P* < 0.01) (Table 1, Figure 1).

In newly diagnosed T2DM group, salivary flow rate, resistin levels in serum and saliva were no significant difference at time points of OGTT. The results suggest that resistin levels in serum and saliva are not affected by eating activity. However, resistin levels in serum were significantly higher than those in saliva (*P* < 0.01) at each time point of OGTT (Table 2).

### 3.2. Correlation Analysis

In newly diagnosed T2DM group, salivary and serum resistin levels were positively correlated at each time point of OGTT. In control group, salivary and serum resistin levels were positively correlated too. When we merged two sets of data for correlation analysis, this conclusion still holds (Table 3).

We also observed a positive correlation of serum and salivary resistin with BMI and HOMA-IR in T2DM group or control group or merged group (Table 4).

## 4. Discussion

Recently, more and more articles report the detection of biomarkers in saliva [20, 21]. Toda et al. suggested salivary adiponectin as a marker of increased risk of noninsulin-dependent diabetes mellitus or cardiovascular disease [22]. Mirco et al. found that salivary leptin is a candidate diagnostic marker in salivary gland tumors [23]. Mamali et al. introduced a method of determining resistin in saliva along with a significant association with serum resistin levels in healthy people [14]. For the first time, we have demonstrated the level of resistin in saliva of newly diagnostic T2DM patients. Saliva resistin levels of T2DM patients are significantly higher than those of non-diabetes mellitus. Resistin levels in saliva are not affected by eating activity (i.e., oral glucose load) and correlated with serum resistin levels at any time point of OGTT. Measurement of resistin in saliva is noninvasive, simple, and easy to multipoint dynamic observation and thus may be an acceptable alternative to serum sampling [24]. Therefore, it could contribute to the elucidation of the physiology and pathological role of resistin in T2DM.

Adipocytokine resistin is a member of the newly discovered family of cysteine-rich protein. Recent data suggest that macrophages are a major source of human resistin. Given the obesity-insulin resistance-inflammation link and convergence of adipocyte and macrophage function, resistin may provide unique insight into links between obesity, inflammation, and metabolic syndrome risk in humans. However, available data regarding the relationship of serum resistin with BMI and insulin resistance are conflicting. Specifically, AI-Sari et al. confirmed a positive correlation of serum resistin levels with BMI [25]. Tokuyama et al. [26] and Fujinami et al. [27] reported that serum resistin levels in diabetic patients were significantly higher than control, and this change was negatively correlated with insulin sensitivity. Our study observed a positive correlation of serum and salivary resistin with BMI and HOMA-IR in T2DM group or control group or merged group (considered T2DM group and control together). This is consistent with the previous findings. Therefore, resistin levels in saliva may be used as a tool to evaluate inflammation/obesity/insulin resistance state for T2DM patients. However, in Mamali et al. research [14], no association of serum and salivary resistin levels with either BMI or insulin sensitivity was reported. They also elaborated the possible reasons of discrepancy, such as the sample size of each study, different hormonal determination methods, and lack of adjustment to the effect of additional variables. Further studies should be pursued in future investigations.

Source of resistin in saliva is not clear to date. Marchetti et al. [28] found that diabetes increased salivary gland basement membrane permeability, allowing serum proteins to saliva by ultrafiltration. However, Carda et al. [29] found that the parotid acinar and interstitial tissue of T2DM patients were rich in lipids, which suggested adipokines in saliva of these people may be secreted by fat cells in the salivary glands. Boström et al. [30] found that the levels of resistin were upregulated locally in the salivary glands and corresponded to the intensity of lymphocytic inflammation in patients with Sjögren's syndrome, which suggested resistin is expressed in the salivary glands of those patients and may be a driving factor of local inflammation. In our study, we found that serum resistin levels were significantly higher than salivary resistin levels of T2DM patients, and the saliva and serum levels are correlated. Furthermore, the fluctuating trend of resistin levels in saliva and blood during OGTT is consistent. Therefore, the source of saliva resistin in newly diagnostic T2DM is mainly derived from blood resistin by ultrafiltration. 

## 5. Conclusions

To sum up, this is the first study to show the analysis of salivary resistin in T2DM. Resistin levels in saliva may be used as a tool to evaluate inflammation/obesity/insulin resistance state for T2DM patients.

## Figures and Tables

**Table 1:** The general information and laboratory data of the new diagnosed type 2 diabetic patients and non-diabetes mellitus.

	Control	T2DM
Participants (M/F)	35 (18/17)	38 (18/20)
Age (years)	43 ± 13	45 ± 10
Body mass index (kg/m^2^)	23.9 ± 3.3	25.5 ± 4.9
Waist circumference (cm)	83.1 ± 6.0	92.2 ± 5.1
Systolic pressure (mmHg)	125.1 ± 12.4	138.0 ± 18.3
Diastolic pressure (mmHg)	78.3 ± 8.2	86.1 ± 9.3
Fasting plasma glucose (mM)	6.1 ± 0.9	7.4 ± 1.7*
HbA1c (%)	6.0 ± 0.6	7.1 ± 0.6*
Fasting insulin (mIU/L)	5.7 ± 2.8	9.2 ± 6.2*
HOMA-IR	3.3 (2.5~4.6)	5.5 (3.6~6.7)*
Fasting serum resistin (ng/mL)	6.1 ± 0.6	12.3 ± 2.7*
Fasting saliva resistin (ng/mL)	1.5 ± 0.3^#^	3.4 ± 0.4^∗#^
Fasting saliva flow rate (mL/min)	2.2 ± 0.8	2.4 ± 1.1

Results are displayed as mean values ± standard deviation. Data of nonnormal distribution were described using the median (M) and quartile (Q1~Q4).

*P < 0.05: comparison of resistin level between T2DM group and non-diabetes mellitus group.

^#^ *P* < 0.01: comparison between serum resistin level and saliva resistin level.

**Table 2:** The general information and laboratory data of the new diagnosed diabetic patients at each time point of OGTT.

OGTT time (hours)	0	1	2	3	*P* value
Participants (M/F)	38 (18/20)	38 (18/20)	38 (18/20)	38 (18/20)	
Plasma glucose (mM)	7.4 ± 1.7	11.8 ± 3.7	17.5 ± 4.6	10.3 ± 2.7	<0.01
Insulin levels (mIU/L)	9.2 ± 6.2	17.3 ± 10.4	40.2 ± 26.1	27.5 ± 16.7	<0.01
Saliva flow rate (mL/min)	2.4 ± 1.1	2.5 ± 0.9	2.6 ± 1.0	2.5 ± 1.2	>0.05
Serum resistin (ng/mL)	12.3 ± 2.7	11.9 ± 4.6	12.4 ± 5.1	11.4 ± 4.1	>0.05
Saliva resistin (ng/mL)	3.4 ± 0.4^#^	3.1 ± 0.7^#^	3.8 ± 1.1^#^	3.9 ± 0.8^#^	>0.05

Results are displayed as mean values ± standard deviation.

^#^ *P* < 0.01: comparison between serum resistin level and saliva resistin level at each time point of OGTT.

**Table 3:** Correlation analysis between serum resistin and salivary resistin levels at each time point of OGTT in T2DM group or fasting state in control group and merged group.

	OGTT 0 hour (T2DM)	OGTT 1 hour (T2DM)	OGTT 2 hours (T2DM)	OGTT 3 hours (T2DM)	Fasting state (control)	Fasting state (all enrolled people)
*r*	0.407	0.222	0.487	0.338	0.233	0.198
*P*	0.000	0.039	0.000	0.015	0.024	0.032

**Table 4:** Correlation coefficients between serum and salivary resistin levels and anthropometric features values.

	Number	Age	Body mass index	Waist circumference	Systolic pressure	Diastolic pressure	Fasting plasma glucose	Fasting insulin	HbA1c	HOMA-IR
T2DM	38									
Serum resistin		0.11	0.48*	0.01	0.41*	0.18	0.08	0.13	0.22*	0.09
Salivary resistin		0.08	0.39*	0.13	0.12	0.07	0.14	0.09	0.31*	0.20*
Control	35									
Serum resistin		0.09	0.38*	0.09	0.18	0.35*	0.37*	0.21	0.13	0.34*
Salivary resistin		0.12	0.17	0.07	0.01	0.21	0.28*	0.13	0.17	0.19
Merged group	73									
Serum resistin		0.07	0.29*	0.11	0.24*	0.30*	0.22	0.41*	0.13	0.35*
Salivary resistin		0.07	0.33*	0.08	0.22*	0.19	0.30*	0.02	0.06	0.29*

*P < 0.05.
